# Supplementary material for: Cannabis Use in Physicians: A Systematic Review and Meta-Analysis
Source: Medicines (Basel). 2023 Apr 27;10(5):29. doi: 10.3390/medicines10050029 (PMC10221702; doi:10.3390/medicines10050029)
Supplement: Supplementary file 1 [file medicines-10-00029-s001.zip › medicines-2317431-supplementary/S1.pdf]

S1. Details for the search strategy used within each database.

Pubmed

((addict*[Title] AND (physician*[Title] OR doctor*[Title]))

OR (("physician impairment"[MH] OR "physician impairment"[TW] OR "impaired physician"[TW] OR "impaired physicians"[TW] OR "impaired doctor"[TW] OR "impaired doctors"[TW] OR "impaired practitioners"[TW] OR "impaired practitioner"[TW] OR "general practice"[MH] OR "general practice"[TW] OR "practice, general"[TW] OR "family doctor "[TW] OR "primary care practitioner "[TW] OR "primary care physician"[TW] OR "primary care physicians"[TW] OR "family medicine"[TW] OR "GP"[TW] OR "FM"[TW] OR "physicians, family"[MH] OR "physicians, family"[TW] OR "family physician"[TW] OR "physician, family"[TW] OR "family physicians"[TW] OR "general practitioners"[MH] OR "general practitioner"[TW] OR "general practitioners"[TW] OR "practitioner, general"[TW] OR "practitioners, general"[TW] OR "general practice physicians"[TW] OR "general practice physician"[TW] OR "general practitioner"[TW] OR "physicians, primary care"[MH] OR "physicians, primary care"[TW] OR "physician, primary care"[TW])))

AND ((marijuana[TW] OR marihuana[TW] OR hashish[TW] OR cannabis[TW] OR cannabis[MH] OR weed[TW] OR cannabi[TW] OR marihuanas[TW] OR ganja[TW] OR bhang[TW] OR hash[TW]))

Filter Language = none
Filter Dates = none

Embase

'cannabis'/exp

AND ('malpractice'/exp OR 'general practitioner'/exp OR ((addict*:ti OR 'drug abuse':ti OR 'substance abuse':ti) AND (doctor*:ti OR practitioner*:ti OR physician*:ti)))

AND [embase]/lim NOT ([embase]/lim AND [medline]/lim)

Cochrane Library

addict*:ti AND (physician*:ti OR doctor*:ti)

OR

"physician impairment" OR "impaired physician" OR "impaired physicians" OR "impaired doctor" OR "impaired doctors" OR "impaired practitioners" OR "impaired practitioner" OR "general practice" OR "practice, general" OR "family doctor " OR "primary care practitioner " OR "primary care physician" OR "primary care physicians" OR "family medicine" OR "GP" OR "FM" OR "physicians, family" OR "family physician" OR "physician, family" OR "family physicians" OR "general practitioner" OR "general practitioners" OR "practitioner, general" OR "practitioners, general" OR "general practice physicians" OR "general practice physician" OR "general practitioner" OR "physicians, primary care" OR "physician, primary care"

AND ((marijuana OR marihuana OR hashish OR cannabis OR weed OR cannabi OR marihuanas OR ganja OR bhang OR hash))

Filter Language = none
Filter Dates = none

ScienceDirect

addict

AND (physician OR doctor)

AND (marijuana OR cannabis)

Filter Language = none
Filter Dates = none

PSYCINFO

addict* AND (physician* OR doctor*)

OR

"physician impairment" OR "impaired physician" OR "impaired physicians" OR "impaired doctor" OR "impaired doctors" OR "impaired practitioners" OR "impaired practitioner" OR "general practice" OR "practice, general" OR "family doctor " OR "primary care practitioner " OR "primary care physician" OR "primary care physicians" OR "family medicine" OR "GP" OR "FM" OR "physicians, family" OR "family physician" OR "physician, family" OR "family physicians" OR "general practitioner" OR "general practitioners" OR "practitioner, general" OR "practitioners, general" OR "general practice physicians" OR "general practice physician" OR "general practitioner" OR "physicians, primary care" OR "physician, primary care"

AND ((marijuana OR marihuana OR hashish OR cannabis OR weed OR cannabi OR marihuanas OR ganja OR bhang OR hash))
