# Supplementary material for: Cannabis Use in Physicians: A Systematic Review and Meta-Analysis
Source: Medicines (Basel). 2023 Apr 27;10(5):29. doi: 10.3390/medicines10050029 (PMC10221702; doi:10.3390/medicines10050029)
Supplement: Supplementary file 1 [file medicines-10-00029-s001.zip › medicines-2317431-supplementary/S8.pptx]

## Slide 1
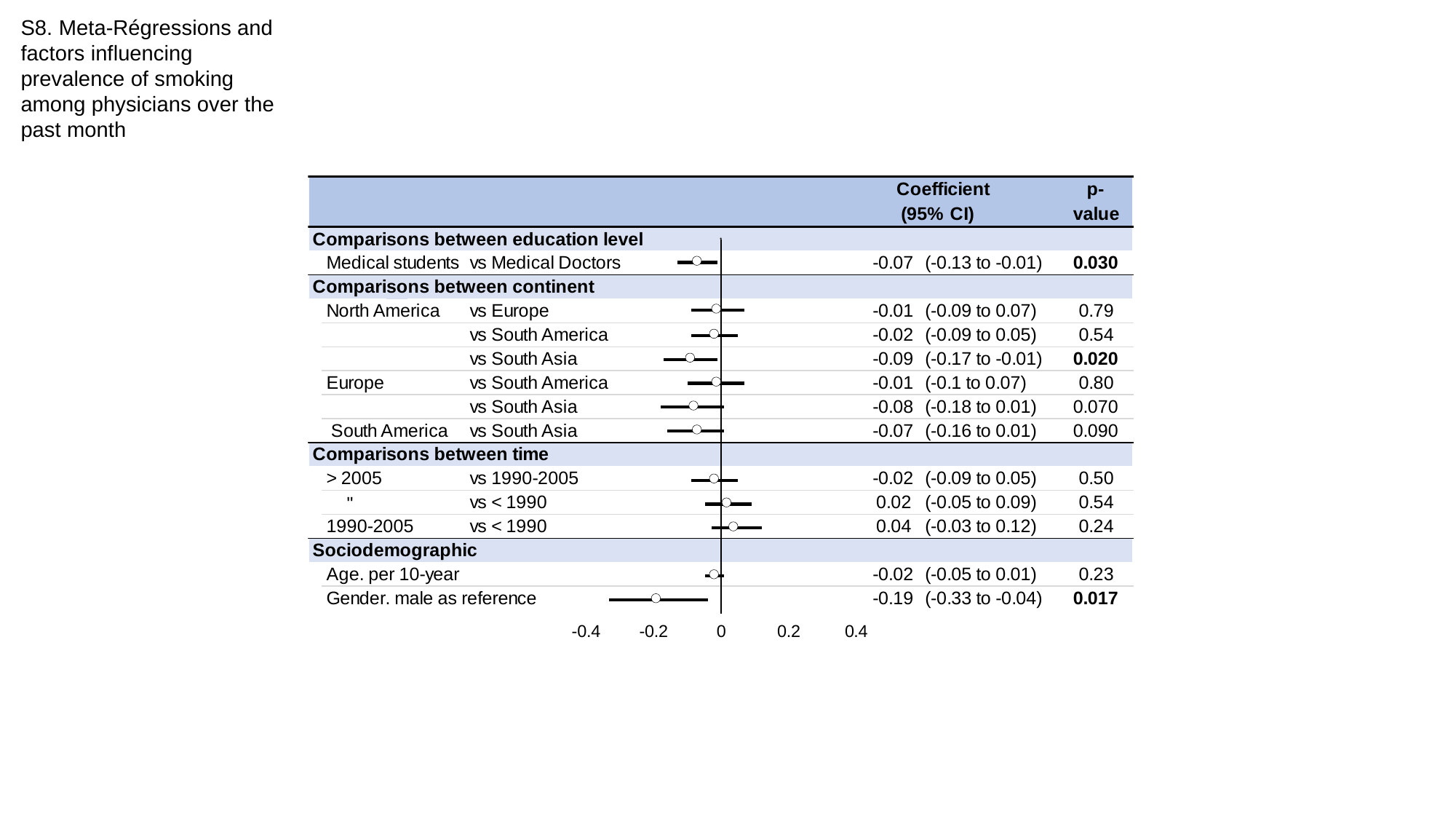

S8. Meta-Régressions and factors influencing prevalence of smoking among physicians over the past month
