# Supplementary figures and images for: Cannabis Use in Physicians: A Systematic Review and Meta-Analysis
Source: Medicines (Basel). 2023 Apr 27;10(5):29. doi: 10.3390/medicines10050029 (PMC10221702; doi:10.3390/medicines10050029)

## Slide 1
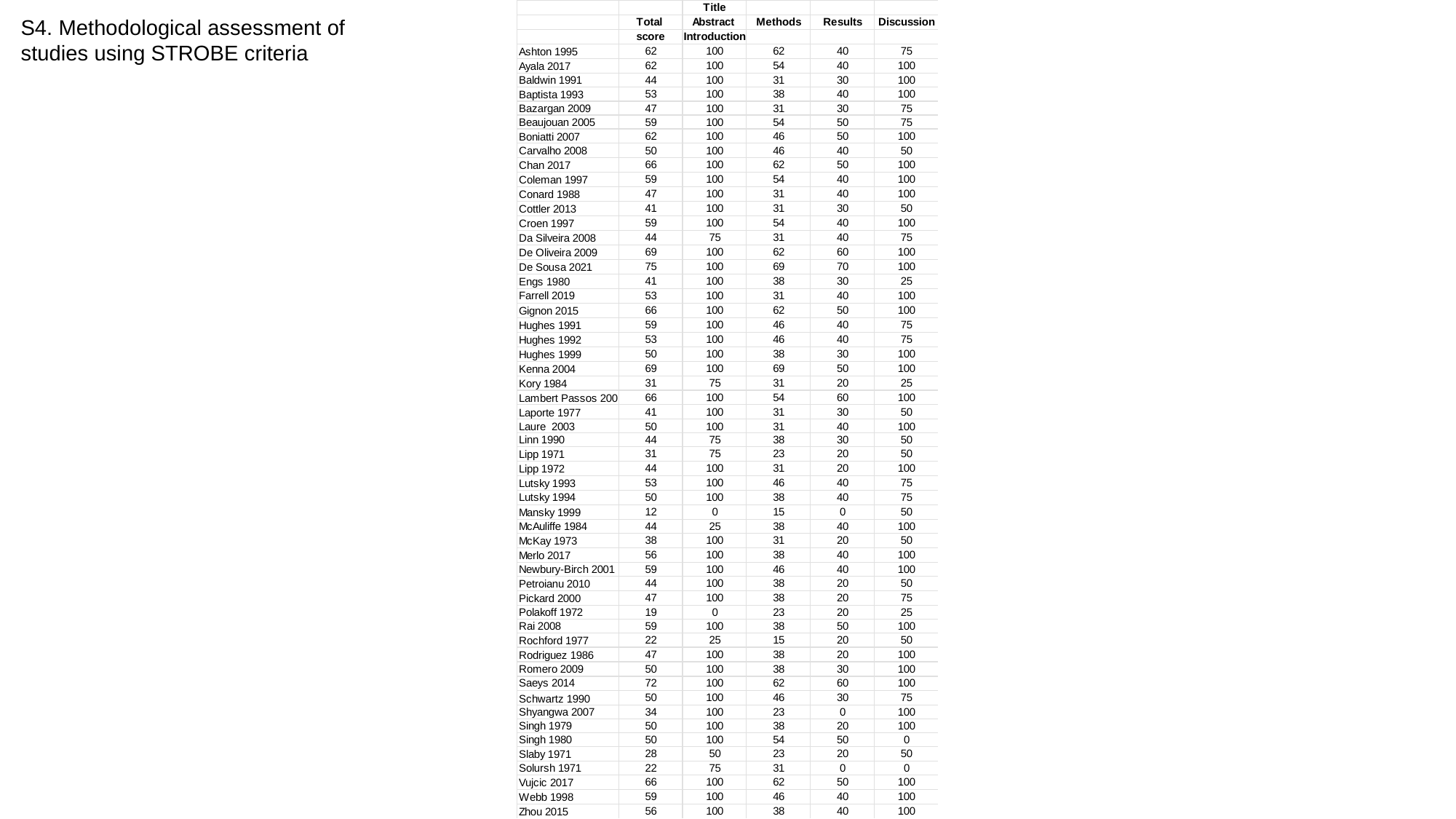

S4. Methodological assessment of studies using STROBE criteria

Supplement: Supplementary file 1 [file medicines-10-00029-s001.zip › medicines-2317431-supplementary/S4.pptx]

## Slide 1
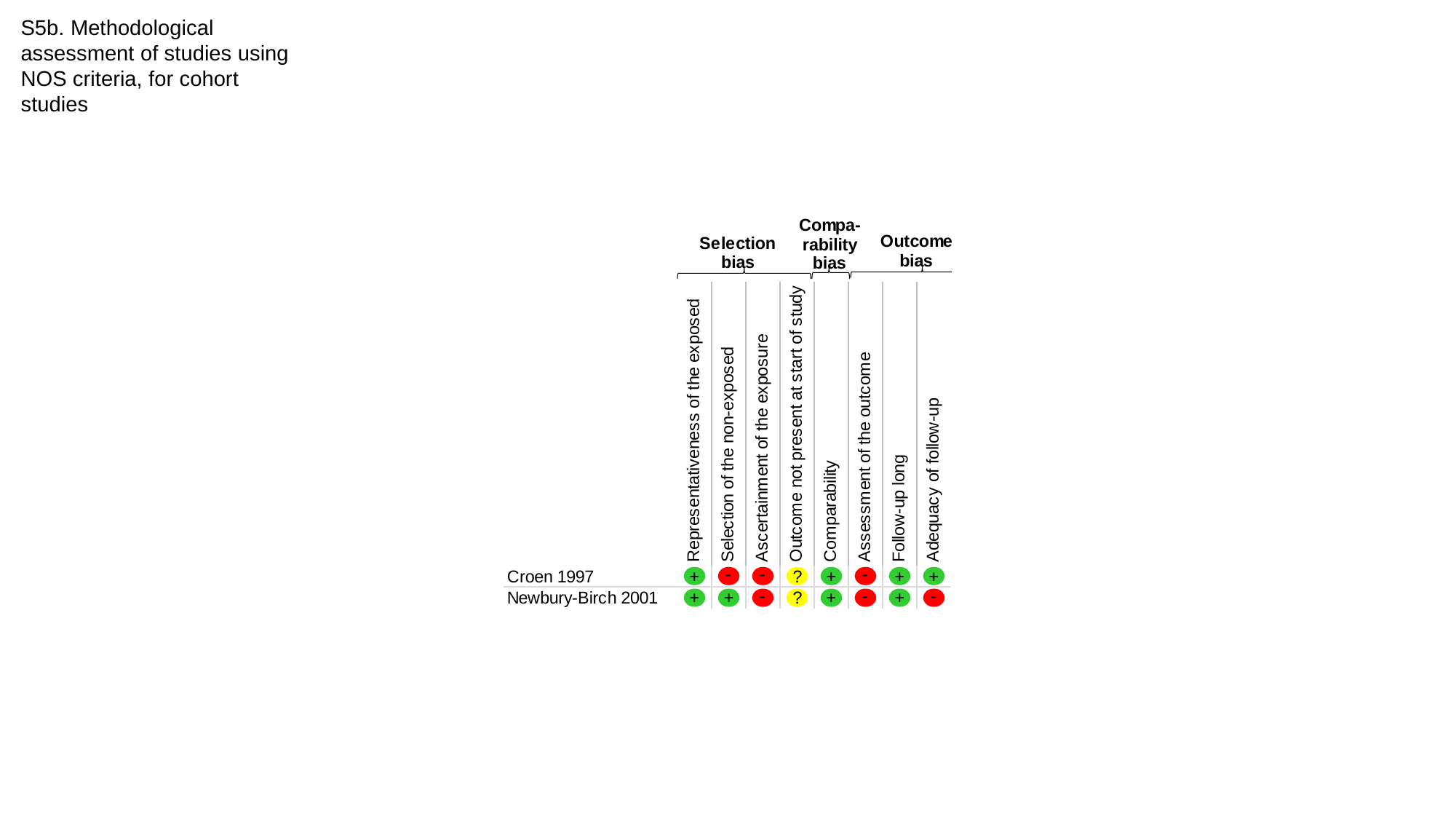

S5b. Methodological assessment of studies using NOS criteria, for cohort studies

Supplement: Supplementary file 1 [file medicines-10-00029-s001.zip › medicines-2317431-supplementary/S5b.pptx]

## Slide 1
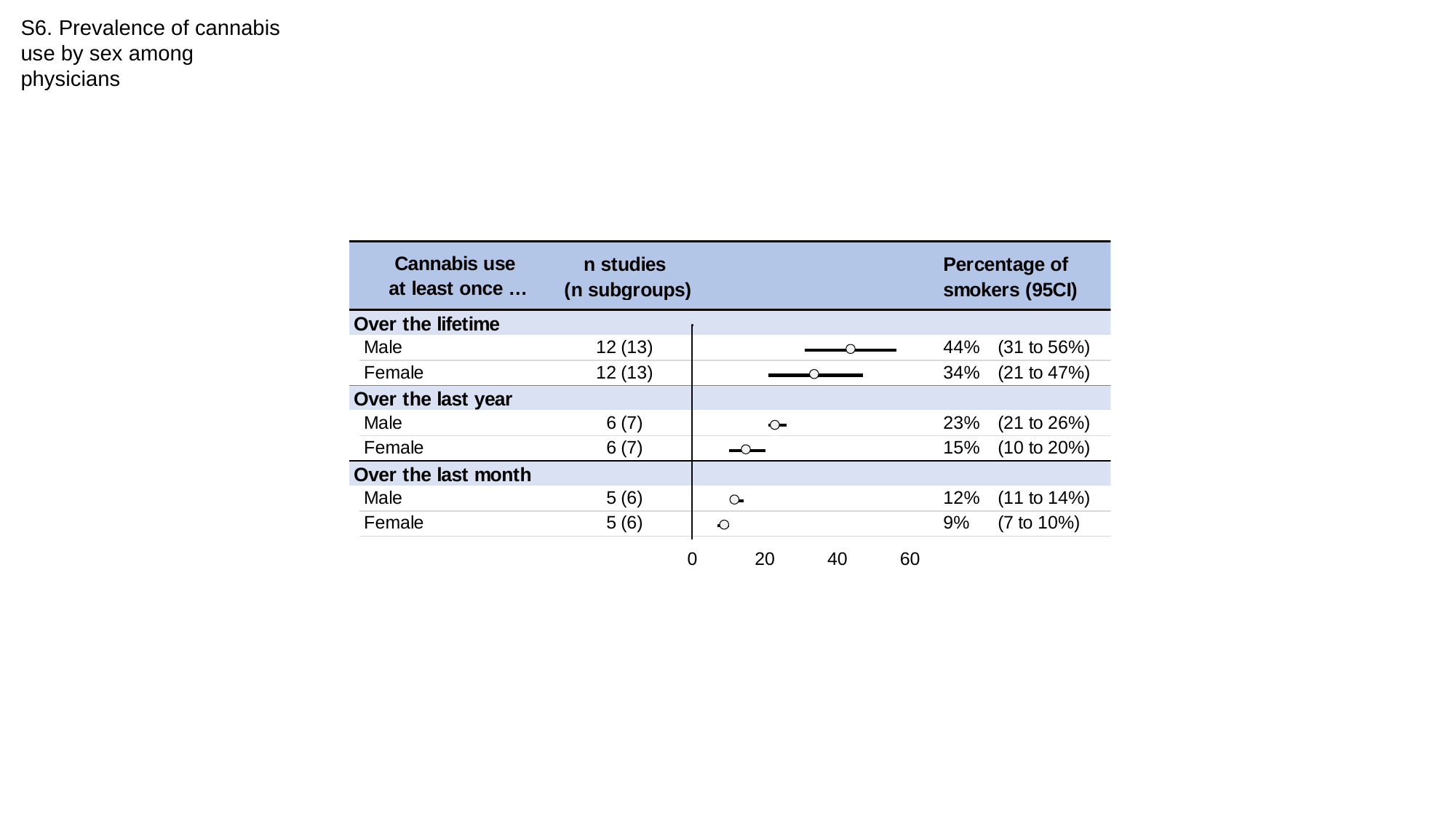

S6. Prevalence of cannabis use by sex among physicians

Supplement: Supplementary file 1 [file medicines-10-00029-s001.zip › medicines-2317431-supplementary/S6.pptx]
